# Supplementary figures and images for: 2020 BioImage Analysis Survey: Community experiences and needs for the future
Source: Biol Imaging. 2021 Nov 12;1:e4. doi: 10.1017/S2633903X21000039 (PMC8982832; doi:10.1017/S2633903X21000039)

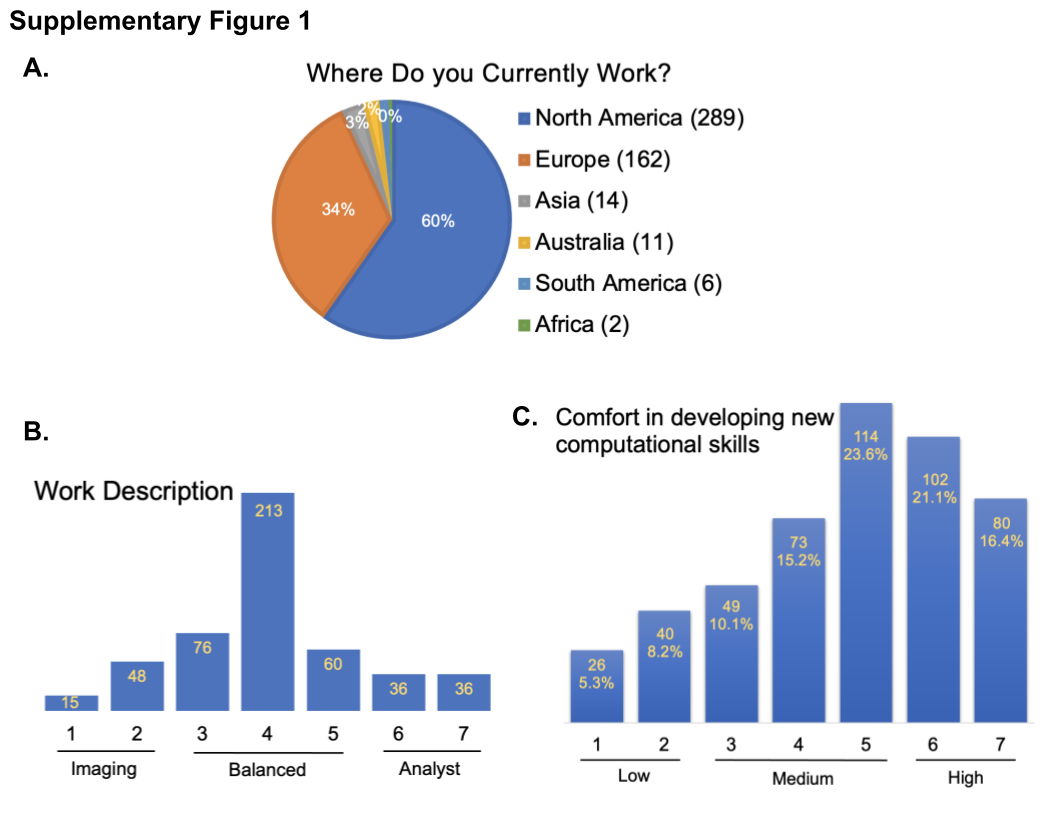

Supplement: Supplementary file 1 [file blgsup.zip › S2633903X21000039sup001.png]

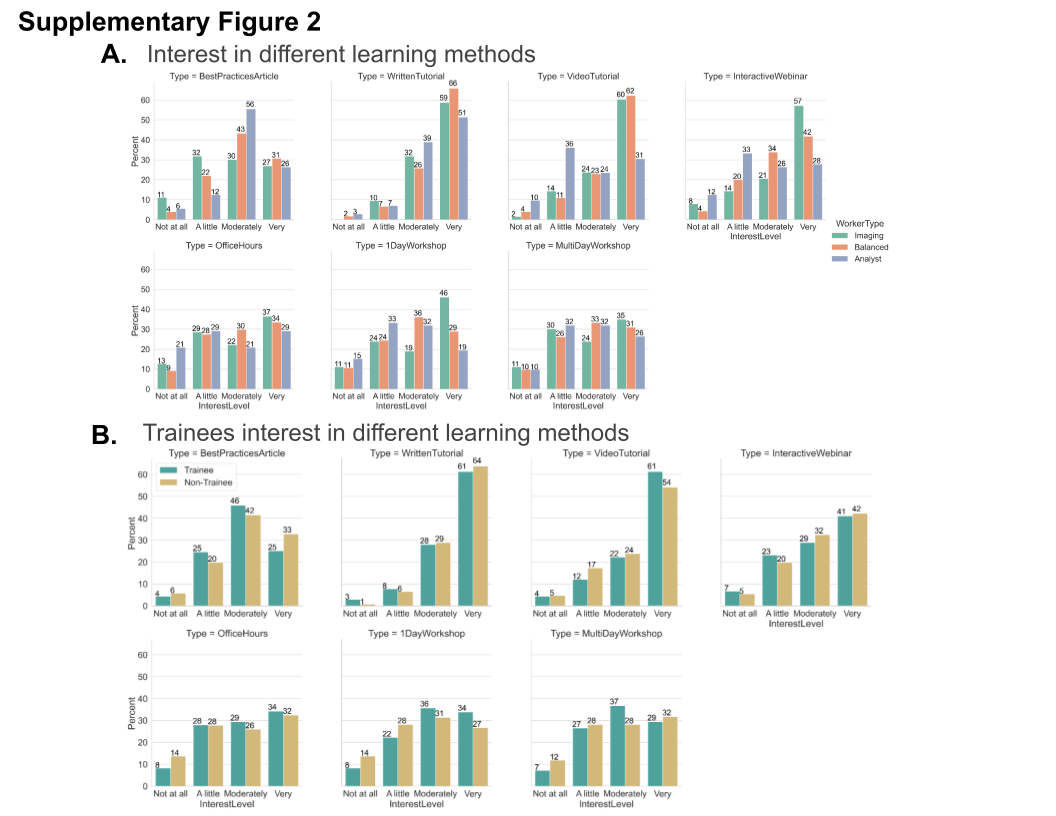

Supplement: Supplementary file 1 [file blgsup.zip › S2633903X21000039sup002.png]

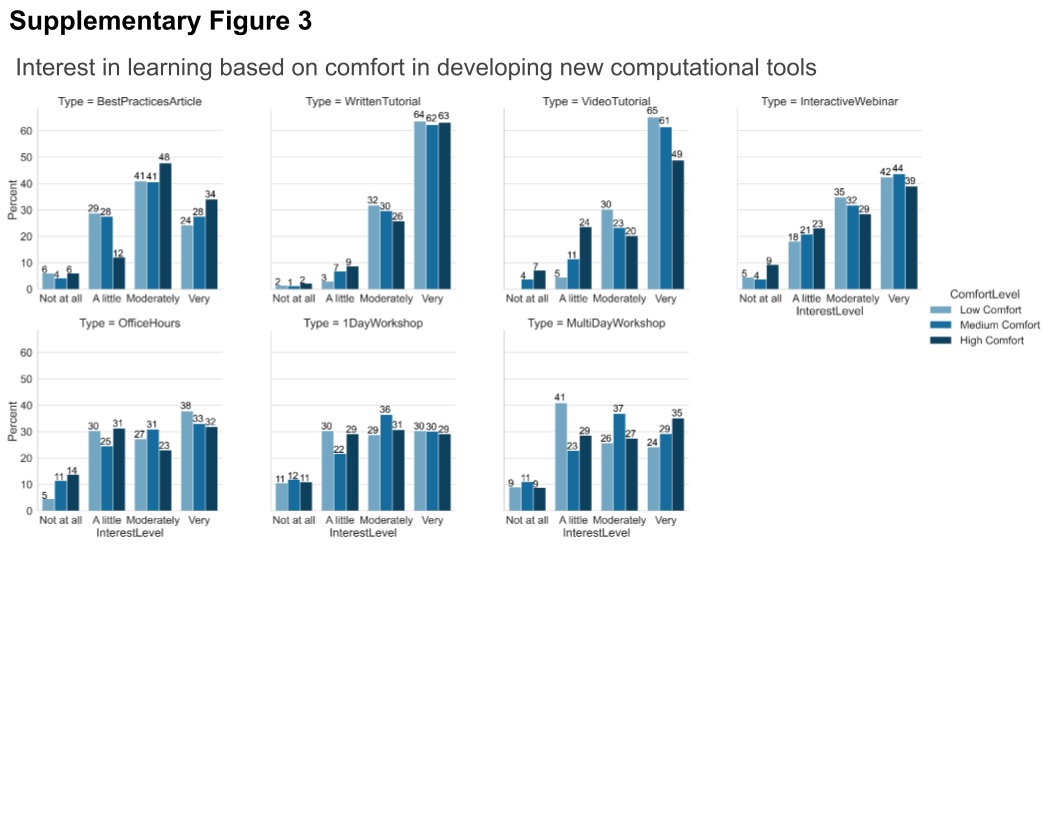

Supplement: Supplementary file 1 [file blgsup.zip › S2633903X21000039sup003.png]

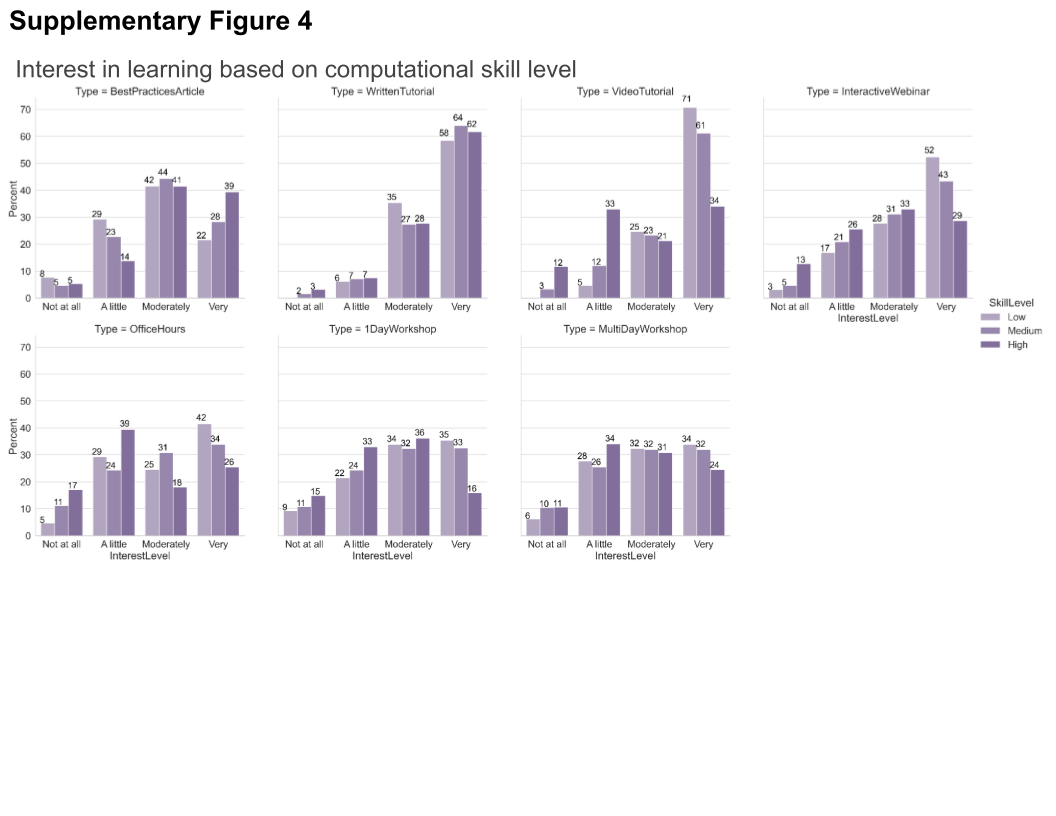

Supplement: Supplementary file 1 [file blgsup.zip › S2633903X21000039sup004.png]
